# Maternal Satisfaction on Delivery Service and Its Associated Factors among Mothers Who Gave Birth in Public Health Facilities of Debre Markos Town, Northwest Ethiopia

**DOI:** 10.1155/2015/460767

**Published:** 2015-08-10

**Authors:** Kurabachew Bitew, Mekonnen Ayichiluhm, Kedir Yimam

**Affiliations:** ^1^Debre Markos Municipality Office, Debre Markos, Ethiopia; ^2^GAMBY College of Medical Sciences, Bahir Dar, Ethiopia; ^3^Department of Public Health, College of Medicine and Health Science, Debre Markos University, P.O. Box 269, Debre Markos, Ethiopia

## Abstract

*Introduction*. The existence of maternal health service does not guarantee its use by women; neither does the use of maternal health service guarantee optimal outcomes for women. The World Health Organization recommends monitoring and evaluation of maternal satisfaction to improve the quality and efficiency of health care during childbirth. Thus, this study aimed at assessing maternal satisfaction on delivery service and factors associated with it. *Methods*. Community based cross-sectional study was conducted in Debre Markos town from March to April 2014. Systematic random sampling technique were used to select 398 mothers who gave birth within one year. The satisfaction of mothers was measured using 19 questions which were adopted from Donabedian quality assessment framework. Binary logistic regression was fitted to identify independent predictors. *Result*. Among mothers, the overall satisfaction on delivery service was found to be 318 (81.7%). Having plan to deliver at health institution (AOR = 3.30, 95% CI: 1.38–7.9) and laboring time of less than six hours (AOR = 4.03, 95% CI: 1.66–9.79) were positively associated with maternal satisfaction on delivery service. Those mothers who gave birth using spontaneous vaginal delivery (AOR = 0.11, 95% CI: 0.023–0.51) were inversely related to maternal satisfaction on delivery service. *Conclusion*. This study revealed that the overall satisfaction of mothers on delivery service was found to be suboptimal. Reasons for delivery visit, duration of labor, and mode of delivery are independent predictors of maternal satisfaction. Thus, there is a need of an intervention on the independent predictors.

## 1. Introduction

Improving maternal health is one of the eight Millennium Development Goals (MDGs) adopted by the international community in 2000. Under MDG5, countries committed to reducing maternal mortality by three quarters between 1990 and 2015 [1]. Globally, maternal mortality is unacceptably high; about 800 women die from pregnancy or child birth related complications around the world every day. Two hundred eighty-seven thousand women died during and following pregnancy and childbirth in 2010; more than half of these deaths occur in Africa. The ratio of maternal mortality in the Sub-Saharan Africa region is one of the highest, reaching 686 per 100,000 live births [2, 3]. In Ethiopia the estimated maternal mortality ratio was 676 per 100,000 live births. Maternal mortality rate among women is 1.14; it represents 30 percent of all deaths for women of age 15–49 [4].

The existence of maternal health service does not guarantee their use by women; neither does the use of maternal health service guarantee optimal out comes for women [2]. The World Health Organization promotes skilled attendance at every birth to reduce maternal mortality and recommends that women's satisfaction be assessed to improve the quality and effectiveness of health care [5]. Patient satisfaction is a subjective and dynamic perception of the extent to which the expected health care is received [6]. It is not important whether the patient is right or wrong, but what is important is how the patient feels [7].

Studies conducted in Dhaka, Bangladesh, and South Australia indicated that the level of maternal satisfaction on delivery care was 92.3% and 86.1%, respectively [8, 9]. However, the level of satisfaction of mothers in African countries is not enough; only 51.9% and 56% of mothers' were satisfied with delivery services in South Africa and Kenya, respectively [10, 11]. Ethiopian studies conducted in Amhara Referral Hospitals and Assela Hospital reported 61.9% and 80.7% satisfaction of mothers on delivery services, respectively [12, 13].

The World Health Organization recommends monitoring and evaluation of maternal satisfaction in public health care sectors to improve the quality and efficiency of health care during pregnancy, childbirth, and puerperium [5]. Maternal caregivers also wish to determine which factors in perinatal care are important for women's satisfaction [14, 15]. Moreover, almost all of the previous studies were hospital based which may create fear on mothers to tell the bad side of the service. Thus, this study aimed at assessing satisfaction on delivery service and its associated factors among mothers who gave delivery in public health facilities of Debre Markos town.

## 2. Methods

### 2.1. Study Design and Study Setting

Community based cross-sectional study design was used to assess level of satisfaction on delivery service and its associated factors among mothers attending delivery room of public health institutions in Debre Markos town from March up to April 2014. The study was conducted in Debre Markos town which is located 300 Km from the capital city of Ethiopia, Addis Ababa, and 276 km from the Amhara region capital, Bahir Dar. There is one referral hospital and three governmental health centers in which all are providing delivery service.

### 2.2. Source and Study Population

All mothers who visited public health institution in Debre Markos town for delivery service were a source population. Those who gave delivery within one year in public health institutions of Debre Markos town were included. And those who were in serious illness and are unable to hear were excluded.

### 2.3. Sample Size Determination and Sampling Procedure

The sample size for this study was determined using single population proportion formula taking the prevalence of delivering mothers satisfied with hospital delivery care service in Amhara region (61.9%) [7]. Considering 95% CI and 5% margin of error and 10% nonresponse rate the final sample size was found to be 398. Systematic random sampling technique with proportional allocation of mothers who gave birth within one year in each kebele of Debre Markos town was used to select the samples of this study.

### 2.4. Variables of the Study

Maternal satisfaction on delivery service was the dependent variable of this study. The independent variables of this study were sociodemographic factors including age, marital status, religion, education, occupation and level of income and obstetric factors including type of delivery, health condition after delivery, ANC follow-ups, duration of labor for last delivery, fetal outcome, age at first pregnancy, duration of time after last delivery, and knowledge on ANC service.

### 2.5. Operational Definitions

The overall satisfaction of mothers was measured based on the answer which they give for question related to satisfaction and if their response is fairly satisfied and very satisfied for 75% of questions or more they were classified as “satisfied” and otherwise they were classified as “unsatisfied.”

### 2.6. Data Collection

The data were collected by structured questionnaire which have three parts. The first part asks about sociodemographic information of mothers and the second part is all about obstetric factors of the mother. Finally, the satisfaction of mothers was measured using 19 questions which were adopted from Donabedian quality assessment framework [16] presented using a 5-point Likert scale ranging from “very dissatisfied” to “very satisfied.” Seven fourth-year public health students as data collectors from Debre Markos University and one public health graduate as supervisor from municipal office were recruited for the data collection process. Then, the questionnaires were administered using house to house visit of seven kebeles.

### 2.7. Data Quality Control

The questionnaire was first prepared in English and translated to Amharic and then translated back to English in order to ensure its consistency. The data collectors and supervisor were given two days of training about the objective of the study and each component of the questionnaire and after the training was given pretest was conducted on 20 respondents from Amanuel town. Principal investigator and supervisor made on the spot checking and reviewed all the completed questionnaires to ensure completeness and consistency of the information collected and immediate action was made.

### 2.8. Data Processing and Analysis

Data were entered and analyzed by using SPSS version 20. Descriptive statistics was computed for the study variables. To identify factors associated with mothers' satisfaction on delivery service binary logistic regression model was fitted. Odds ratio and 95% confidence interval were used to determine the strength of association between dependent and independent variables.

### 2.9. Ethical Consideration

Ethical clearance was obtained from the Ethical Review Board of Debre Markos University. Communication with the kebele administrators was made through formal letter obtained from Debre Markos University. After the purpose and objective of the study were informed, verbal consent was obtained from each study participant. Participants were also informed that participation will be on voluntary basis and they can withdraw from the study at any time. Confidentiality of information provided by study subjects was also protected by making the data collection procedure anonymous.

## 3. Result

### 3.1. Sociodemographic Characteristics of Mothers

The survey included 398 mothers, of these 9 (2.61%) were not found at home on three consecutive visits and registered as nonresponse. Among respondents, 182 (46.8%) of them belonged to the age group of 26–35, followed by 15–25 age groups accounting for 167 (42.9%) of respondents, and the remaining 40 (10.3%) of them belonged to the age group of 36–45. The mean (±SD) age of the respondents was 27.4 (5.3) years; the minimum age was 18 years and the maximum age was 43 years. The majority of the respondents (376 (96.7%)) were followers of orthodox Christianity and the remaining were Muslims (13 (3.3%)) (Table 1).

Regarding educational status of mothers, 108 (27.8%) of them were at high school or preparatory level (9–12 grades) and 98 (25.2%) of them were at primary school level. About 371 (95.4%) of mothers were married and the remaining 18 (4.6%) of them were single or divorced. One hundred eighty-six (47.8%) of them were housewives in occupational status and 148 (38%) of them reported average monthly household income of less than 999 Ethiopian Birr (Table 1).

### 3.2. Obstetric Characteristics of Mothers

About 322 (82.8%) of mothers reported that their first pregnancy was conceived at 15–25 years of age. Three hundred fifty-five (91.3%) of pregnancies were wanted and 378 (79.4) of mothers had at least one ANC visit for the current pregnancy. The majority (363 (93.3%)) of mothers reported that they had plan to deliver at health institutions and the remaining 26 (6.7%) of them were delivered in the health institutions because of difficulty to deliver at home. Among mothers, 265 (68.6%) of them have reported that the labor stayed for less than 6 hours and assisted vaginal delivery was the mode of delivery for 189 (48.6%) of them. Health complication after delivery was experienced by 364 (93.6%) of mothers and the fetal outcome of almost all 388 (99%) pregnancies was live birth (Table 2).

### 3.3. Maternal Satisfaction on Delivery Service

**Figure 1:**
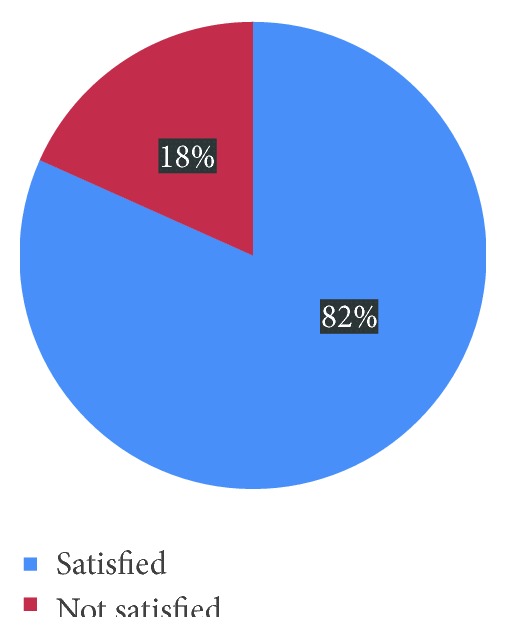
Prevalence of maternal satisfaction on delivery service among mothers who gave birth within one year in Debre Markos town, Northwest Ethiopia, 2015.

Among mothers, more than 95% of mothers were satisfied with the helpfulness of staff and health facility waiting time to be seen by health workers. And less than 85% of mothers were satisfied by accessibility of transportation and accessibility and cleanliness of toilet. Regarding their interaction with staff, more than 95% of reported that they were satisfied with use of delivery position of their choice and communication among health care providers. However, 73% and 57% of mothers were satisfied with explanation of health providers about the drugs prescribed and their side effects, respectively. About 98% and 87% of respondents were satisfied with assurance of privacy and respecting of social norms and privacy, respectively (Table 3). Among 398 mothers who gave birth within one year, the overall satisfaction of them on delivery service was found to be 318 (81.7%) (Figure 1).

### 3.4. Factors Associated with Maternal Satisfaction on Delivery Service

In multivariate analysis, having plan to deliver at health institution (AOR = 3.30, 95% CI: 1.38–7.9) and laboring time of less than six hours (AOR = 4.03, 95% CI: 1.66–9.79) were significantly and positively associated with maternal satisfaction on delivery service. The likelihood of maternal satisfaction on delivery service was lower (AOR = 0.11, 95% CI: 0.023–0.51) among mothers who experienced spontaneous vaginal delivery as compared to mothers who experienced cesarean section (Table 4).

## 4. Discussion

In this study, the overall satisfaction of mothers on delivery service was found to be 81.7% which was in line with the study conducted in Wolayita Zone (82.9%), Jimma (77%), and Assela Hospital (80.7%) [9, 12, 17]. However, this finding was higher (61.9%) than the study which was conducted in Amhara Referral Hospitals. The difference with the above finding could be explained by a real difference in the quality of services provided (as Debre Markos Referral Hospital received national quality award by MOH in 2014), expectation of mothers, or the type of health facilities [13]. In addition, two of the three referral hospitals selected in Amhara region study were teaching based which may increase their dissatisfaction due to reasons such as repeated vaginal examination and absence of privacy.

Having plan to deliver at health facility was significantly and positively associated with maternal satisfaction on delivery services. This finding was similar with the study conducted in Assela Hospital and other foreign literatures. The literatures showed that clients had various expectations about hospital delivery that influenced their perception of care. These expectations were based on their own past experiences in a hospital facility, experiences of friends and relations in a hospital facility, myths about procedures in the hospital and societal values, and perception of an assisted delivery [12, 18].

In this study, laboring time of mothers was significantly associated with satisfaction of mothers on delivery services. Similarly, a study conducted in Wolayita Zone showed that the satisfaction of mothers who stayed on labor pain for less than a sunset was higher. One study in North Gondar Zone also indicated that suffering with complication and having long labor influence the satisfaction of mothers on health institution delivery service [19, 20]. A randomized controlled trial of active management of labor conducted in Auckland, New Zealand, reported that the practice was related to reduction of labor time and a postpartum report of longer labor than expected was strongly associated with reduction of maternal satisfaction [21, 22].

This study demonstrated that spontaneous vaginal delivery was significantly associated with reduction of satisfaction on delivery care. This finding was unexpected and not in line with report of other similar studies [23, 24]. Several other studies also failed to detect difference in level of maternal satisfaction among those who have different mode of delivery [13, 22]. As in Ethiopia most of cesarean section deliveries were conducted at emergency situation, saving their baby in that situation may change their perception towards the facility which might be the possible explanation for the finding.

Absence of inferring causality due to cross-sectional nature of the study and the presence of recall bias due to long time lapse after childbirth are among the limitations of this study. Regarding the strengths of this study, community based nature of this study also minimizes social desirability bias.

## 5. Conclusion

This study revealed that the overall satisfaction of mothers on delivery service was found to be suboptimal. However, there is still much work needed in the domain of interaction of mothers with health care providers. Reasons for delivery visit, duration of labor, and mode of delivery are independent predictors of maternal satisfaction. Thus, there is a need of an intervention on the independent predictors.

## Figures and Tables

**Table 1:** Sociodemographic characteristics of mothers who gave birth within one year in Debre Markos town, Northwest Ethiopia, 2014.

Variables	Frequency	Percentage (%)
Age		
18–25	167	42.9
26–35	182	46.8
36–45	40	10.3
Religion		
Orthodox Christian	376	96.7
Muslim	13	3.3
Educational status		
Unable to read and write	60	15.4
Able to read and write only	29	7.5
Primary school (1–8th)	98	25.2
High school or preparatory (9–12th)	108	27.8
Certificate and above	94	24.2
Marital status		
Married	371	95.4
Single	10	2.6
Divorced	8	2.1
Occupational status		
Housewife	186	47.8
Merchant	92	23.7
Government employee	57	14.7
Nongovernment employer	20	5.1
Daily laborer	18	4.6
Student	16	4.1
Average household monthly income		
>999	148	38
999–1399	83	21.3
1400–1999	44	11.3
2000–2599	45	11.6
2600–3399	41	10.5
≥3400	28	7.2

**Table 2:** Obstetrics characteristics among mothers who gave birth within one year in Debre Markos town, Northwest Ethiopia, 2014.

Variables	Frequency	Percentage (%)
Age at first pregnancy		
15–25	322	82.8
26–35	67	17.2
Parity		
Two	182	46.8
Three	165	42.4
Four or more	42	10.8
Wanted status of pregnancy		
Yes	355	91.3
No	34	8.7
Number of ANC visits		
One	378	79.4
Two	60	15.4
Three	11	2.8
Four	9	2.3
Reason for visit		
Planned	363	93.3
Referral	26	6.7
Duration of labor		
<6 hours	265	68.1
6–12 hours	91	31.5
12–24 hours	17	4.3
Above 24 hours	16	4.1
Type of delivery		
Assisted delivery	189	48.6
Spontaneous vaginal delivery	168	43.2
Cesarean section	32	8.2
Condition of mother after delivery		
Without complication	364	93.6
With complication	25	6.4
Outcome of pregnancy		
Lived	385	99
Died	4	1.0
Time passed after delivery		
≤6 months	212	54.5
>6 months	177	45.5

**Table 3:** Satisfaction on health facility and staff related variables among mothers who gave birth in public facilities of Debre Markos town, Northwest Ethiopia, 2014.

Domains	Variables	Satisfaction	
		Yes, *n* (%)	No, *n* (%)
Physical and staff accessibility	Examination area cleanliness and comfort	373 (95.9)	16 (4.1%)
	Restfulness of the rooms of the facility	360 (92.5)	29 (7.5)
	Overall cleanness of the facility	345 (88.7)	44 (11.3)
	Availability of transportation	326 (83.8)	63 (16.2)
	Supplies of basic drugs and equipment	349 (89.7)	40 (10.3)
	Accessibility and cleanness of toilets and/or shower	324 (83.3)	65 (16.7)
	Waiting time to be seen by health worker	372 (95.6)	17 (4.4)

Interaction with providers and staff of the facility	Helpfulness of staff	377 (96.9)	12 (3.1)
	Explanation about the treatment given	359 (92.3)	30 (7.7)
	Communication between health care providers	383 (98.5)	6 (1.5)
	Opportunity to ask about the treatment	343 (88.2)	46 (11.8)
	Involvement of patient in decision making	305 (78.4)	85 (21.6)
	Willingness of staff to hear patient problems	368 (94.6)	21 (5.4)
	Explanation about the drugs prescribed	284 (73)	105 (27)
	Explanation about the side effects of drugs	222 (57.1)	167 (42.9)
	Delivery position of patient choice	372 (95.6)	14 (4.4)

Provision of respect and privacy	Respect and assurance of privacy	380 (97.7)	9 (2.3)
	Respect of social norms and values	329 (84.6)	60 (15.4)
	Equal treatment of people	339 (87.1)	50 (12.9)

**Table 4:** Factors associated with maternal satisfaction on delivery service among those who gave birth within one year in Debre Markos town, Northwest Ethiopia, 2014.

Variables	Maternal satisfaction		Crude OR (95% CI)	Adjusted OR (95% CI)
	Satisfied	Unsatisfied		
Reason for delivery visit				
Planned	302 (83.2%)	61 (16.8%)	3.09 (1.34–7.143)	**3.30 (1.38–97.9)** ^**∗∗**^
Referred	16 (61.5%)	10 (38.5%)	1	1
Laboring time				
<6 hours	227 (85.7%)	38 (14.3%)	2.60 (1.15–5.89)	**4.03 (1.66–9.79)** ^**∗∗**^
6–12 hours	68 (74.7%)	23 (25.3%)	1.29 (0.53–3.10)	1.45 (0.57–3.68)
Above 12 hours	23 (69.7%)	10 (30.3%)	1	1
Mode of delivery				
Spontaneous vaginal	127 (75.6%)	41 (24.4%)	0.21 (0.05–0.90)	**0.11 (0.023–0.51)** ^**∗∗**^
Assisted delivery	161 (85.2%)	28 (14.8%)	0.38 (0.09–1.70)	0.27 (0.06–1.24)
Cesarean section	30 (93.75)	2 (6.25%)	1	1

^*∗∗*^ *p*-value less than 0.001.
